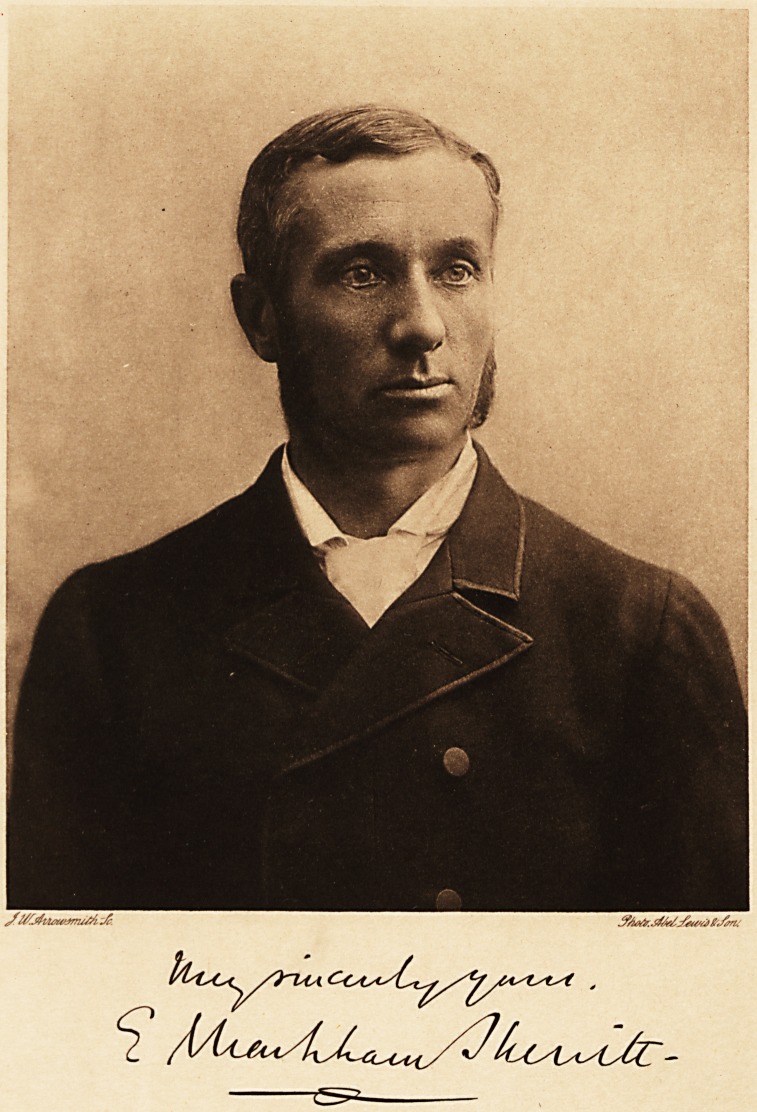# Edward Markham Skerritt, M.D. Lond., F.R.C.P.

**Published:** 1907-06

**Authors:** 


E. Markham Skerritt

### The Bristol
### Medico-Chirurgical Journal.

*"Scire est nescire, nisi id me

Scire alius scireet."*

JUNE, 1907.

### Obituary Notice.

### EDWARD MARKHAM SKERRITT, M.D., F.R.C.P.

Thou whom none knoweth, yet they lie
Who say Thou art not, speak with me!
I am because Thou bidst me be,
And when Thou bidst me, I must die. . . .

Oh, rend the Heaven! Break up the height,
The depth, between Thy works and Thee!
Tear off the veil, that Earth may see
The fount of good, the judge of right![1]—BOURDILLON.

It is only human to desire to record some memorial of a man we have lost, especially when he has been associated with us in many years of similar work, and when we so well know that his life was a model from which we may ourselves draw many useful lessons. In the twenty-five years of the life of this *Journal* we have had many occasions to give an "In memoriam" record of the lives of those who have gone before. Our readers will remember Augustin Prichard, Edward Long Fox, William Johnstone Fyffe, Joseph Griffiths Swayne, Henry Marshall, Aust Lawrence, and the first

[1] *Monthly Review*, March, 1907.

editor of this Journal, James Greig Smith, whose life, all too short, was terminated somewhat abruptly in the midst of active work by an attack of pneumonia. And now we have to lament the death of Edward Markham Skerritt, also prematurely cut off by pneumonia at perhaps the most useful period of his career.

We cherish the memory of our "mighty dead," but their message to us is that it is the business of man to keep things going; no one of us is indispensable, but, nevertheless, the best of us are much missed at the time of departure. He who has so lived as to "leave the world a little better than he found it," must of necessity leave a gap when his chair becomes vacant.

That such a gap followed the death of Dr. Skerritt was shown by the striking demonstration at the funeral, which took place on Thursday, May 2nd, 1907, when the presence at St. Paul's Church of a very large number of mourners, including a large proportion of his own profession—by whom he was so much esteemed—gave the most pronounced testimony to the widespread feeling of regret that his life's work had ended. He was buried at Redland Green amidst every manifestation of sadness and regret, expressed not only by his own immediate friends, professional colleagues and patients, but by representatives of the various public bodies with which his work had been associated.

Edward Markham Skerritt was born at Chelsea on December 30th, 1848, and he died on Monday, April 29th, 1907, at the comparatively early age of 59. His education commenced at Mill Hill School, but afterwards he was sent to Amersham Hall School, where he found as school-fellows many associates who have risen to considerable distinction in various walks of life. His early education appears to have been of a robust and vigorous if not of an ascetic type, and he ever after lived a most active, strenuous career, never sparing himself or taking rest and quiet. On leaving school he matriculated at the University of London (1866), and graduated as B.A. Lond. in 1868. In the following year he passed the Preliminary Scientific M.B. Examination, and then entered as a medical student at University College, London, at a time when this school was in a very vigorous state, and attracted a large proportion of the ablest students in London, many of whom have come to the front and are occupying leading positions in London and the provinces. With such competitors Skerritt easily held his own; he won many prizes and class distinctions, and when he passed the examinations for the M.B., B.S. he obtained the University gold medals for Physiology, Medicine, and Obstetric Medicine. In 1874 he obtained the degree of M.D. Lond. As a student he was noted for his unflagging industry, and a methodical habit which enabled him to acquire knowledge easily and to learn accurately. Although reserved, he was much liked by his fellow students, who admired his ability, and were attracted by his simple straightforwardness. As a mark of the success of his student's course he was elected a Fellow of his College, and he obtained the Atkinson-Morley surgical scholarship, which was the highest surgical distinction open to him. As Sir John Erichsen's house surgeon, and Sir William Jenner's house physician, he had the fullest opportunity of gaining the soundest medical and surgical skill, so that by his own natural ability, and by the best possible training, it was felt that Skerritt had laid an excellent foundation for a very distinguished career. It was in 1875 that a vacancy for physician—caused by the death of Dr. Samuel Martyn—at the Bristol General Hospital gave the opportunity which the electors wisely accepted when they appointed him to serve in an institution which for thirty-two years since has gained so much from his painstaking and skilful work as physician. He became M.R.C.P. in 1876, was elected a Fellow in 1885, and afterwards a member of the Council of the Royal College of Physicians of London.

As a clinical teacher he will long be missed. His instruction was good, and he was earnest in imparting it. Many generations of old Bristol students must have a grateful recollection of the benefits they have themselves obtained by his example as well as by precept. As Joint Lecturer and later Professor of Medicine (this course having been always divided between a member of the staff of the Infirmary and one from the Hospital) he was methodical and clear; he devoted especial attention to diseases of the heart and lungs, and these lectures, with a course on general febrile maladies, usually occupied the whole of the time available.

For many years he acted as Secretary, and afterwards became the first Dean of the Medical Faculty in the University College. A colleague writes, "These offices were no sinecure, and gave him an enormous amount of work, as the negotiations for incorporation extended over some years. . . . Dr. Skerritt was *ex officio* a member of the Council of the College, and in all matters medical was the authority; but his knowledge of business, his sound judgment and shrewd common sense, made him a valuable member of the Council in all that related to the welfare of the College." The engineering Chairman of the Council once remarked to the writer that Skerritt appeared to be "a very level-headed person;" and so he was, for he had a wholesome detestation of fads of all descriptions. It was felt that he was not only a skilled physician, but a wise counsellor, a man of sterling uprightness, and of unimpeachable sincerity, essentially a man to be trusted.

He held in turn all the offices which the local profession had to offer. He never declined anything which appeared to be his duty, or in which he could do good service. As Secretary for many years of the Bath and Bristol Branch, as President of the Branch, as a member of the central Council of the Association, as Treasurer of the Association in 1902, and Vice-President in 1904, he was a mainstay of the British Medical Association, and during the last few years, when intricate details under the new constitution have been much discussed, Skerritt's knowledge and clear judgment have been invaluable. Others have written on these points in the *Journal* of the Association (May 11th, p. 1159), and with regrets that "his place in our Council Chamber is empty, and with sorrowful hearts we say farewell to one who has left us before his time."

Skerritt was one of the very early members of the Bristol Medico-Chirurgical Society, and was rarely absent from its monthly meeting. He was President in 1892–3, and then gave a very characteristic presidential address on "The Teachings of Failure." Many other papers and addresses have been published from time to time in this *Journal,* and it will be generally admitted that Skerritt never wrote for the sake of writing, and that whatever he wrote is worth reading. He was not a voluminous writer, but he always had an object in his writing, and expressed himself clearly and concisely. His Bradshaw Lecture, given before the Royal College of Physicians in 1897, is an excellent outline of things which had passed through his mind for many years, and in which he held very definite views. He had no great ambition to be known as an author; his time was actively employed in other ways. He was no advocate of a gospel of inactivity, but his powers were commonly expended in other fields.

The Queen Victoria Jubilee Convalescent Home has, from its commencement, derived the advantage of Dr. Skerritt's presence on its Board of Governors, and his work there is shown by the resolution adopted by them at a meeting on May 17th, as follows: "The Governors of the Convalescent Home desire to place on record their sense of the great loss the City and many of its most useful institutions has incurred in the removal of one whose remarkable abilities were only equalled by his kindness of heart and his readiness to serve others. They record especially their appreciation of the interest he took in the founding of this Home, the value of his aid in arranging all its organisation, and the constant service he rendered in its management."

Another institution which owes much to his services is the Winsley Sanatorium for the poorer consumptives of the three neighbouring counties. He was a member of the original executive committee, and he took a leading part in the initiation and furtherance of a method of treatment which his long experience of chest diseases had shown to be most desirable. It was a satisfaction to him to feel that this Institution is now in good working order, and is becoming more and more increasingly useful.

With one exception he was the oldest member of the Medical Reading Society, and he was present at the centenary meeting, when eighteen past and present members met at dinner on April 3rd. He was then in good health and spirits, looking strong and wiry as usual, with a good expectation of a long continued career of useful activity. The monthly meetings of this Society have conduced much to the furtherance of good fellowship, and Skerritt's presence has doubtless tended towards the diminution of such professional jealousy and rivalry as at times exists amongst many professional communities. Skerritt was elected a member of this little Society in 1876, so that he had assisted in over one-third of its centenarian life.

With regard to the social characteristics of Skerritt, the following personal appreciation is written by one of his most intimate friends, Dr. Barclay J. Baron :—

"My friendship with Edward Markham Skerritt began in 1883, when, as a new-comer to Clifton and a near neighbour, I called on him. He was courteous and kind, as was his wont, but, more important, he was distinctly encouraging as to my prospects of practice in Bristol. This faculty of encouraging younger members of the profession in times of difficulty was a trait in his character of peculiar value to many of us. He invited me to go round the wards of the General Hospital with him, and of this invitation I availed myself, during the next year, on many occasions. I profited greatly by the clear, logical way in which he arrived at a diagnosis. I was struck by his faculty for deducing feasible theories as to the causation of disease from ascertainable facts, and in particular by the simplicity of his therapeutic measures. Markham Skerritt was no more attracted by newly introduced, well advertised or fashionable drugs than he was by the change of fashions in millinery, and I feel certain that in this respect his influence as a physician was of a decidedly beneficial character. At this time he prescribed alcohol for patients in smaller quantities than most men, and as his experience increased he prescribed it less and less, until, in his last days it certainly occupied an unimportant place in his medical armamentarium. His great reliance was on rest and warmth, on *vis medicatrix naturæ* rather than on drugs.

"In 1884 my humble dwelling was visited one evening by Skerritt and Aust Lawrence, and the greeting of the former was 'Le roi est mort: vive le roi!' This meant that the late Dr.

Burder had presented his resignation of the physiciancy of the Bristol General Hospital to his colleagues, and Skerritt asked me if I would become a candidate for the vacant post. Largely owing to his support I was appointed without contest, and thus became his Hospital colleague. I had by this time formed a very high opinion of my new friend, but I had never seen him in counsel with other senior members of the profession until the first staff meeting took place. I was greatly interested in finding that my estimate of him was that held by all his colleagues, and if ever a difference of opinion arose Markham Skerritt almost invariably induced the staff to adopt the views which he himself held. In debate he was certainly very strong, because he never placed himself in a position of antagonism to others without having first satisfied himself that he was right, and with rare ability he never under-estimated the strength of his opponents' attack, and so was fully prepared to meet and overwhelm it.

"Others have spoken to his Hospital and University College career, and to his sterling value to practitioners in consultation. As a consultant, as indeed in other relations of life, Skerritt listened rather than talked. He was distinctly reserved, and, indeed, many patients would have liked him to unbend and say more. In point of fact, he was essentially a home-loving man. He donned a dress-coat unwillingly, and was seen but little at clubs and dinners ; but he received his guests in his own house, at all times, with the refined hospitality of a large heart. Those of us who knew him intimately in Clifton found that he very frequently relaxed the stiffness of his manner, and was full of humour and good fellowship. But to see Markham Skerritt full of the love of life, really humorous and genial, one must have done as I did, gone down and stayed with him on Dartmoor or Exmoor. When I first visited him at Throwleigh, near Chagford, I was met at the farmhouse gate by him with no hat, with certainly no waistcoat, I believe without a necktie. He had then not quite developed his intense devotion to open air living, but he had at least joined the 'hatless brigade.' It was a charming picture of family happiness which greeted me as he, his wife, his daughter and I wandered over those grand old moors. He was intensely observant of the ever-changing beauties of flower and insect life, sunshine and cloud, and deeply impressed by the wonderful solitude of the Devon Tors. For his was indeed a very simple, natural mind, and whilst he pretended to no profound knowledge of Botany or Entomology, he lived his days of leisure on the countryside with a spirit very responsive to its open secret.

"On another occasion I visited him at Avill, near Dunster, whither he was in the habit of taking horses and hunting with the Devon and Somerset Staghounds. By this time he had become a great devotee of the open air, and it was his delight for us all to have breakfast in front of the farmhouse door at a table where a robin, which he had induced to recognise us as friends, came regularly to feed from our plates. On one occasion Aust Lawrence was also a guest, and on the cold days—which sometimes set in at the end of August—it was a constant source of entertainment to watch him (for he hated too much cold air) taking every opportunity of our host's temporary absence from the room to shut the casement windows, and to see Skerritt on his return, finding that the curtains were not properly blowing about, open the windows again with distinct rumblings of wrath at the stupidity of servant girls who did not appreciate their privilege of living in God's air.

"Whatever Skerritt did he did with all his might, and hunting was no exception to the rule. I have known him on horseback from early morning till evening under a blazing sun, with no more refreshment than a few raisins and a little chocolate, and his 'pistol' full of cold tea. He never, with that modesty we all know was so deeply ingrained in his nature, talked much of wonderful runs at which he had been present; but once a well-known sportsman, comparing him with another hunting doctor, said: '—'s heart is as big as a threepenny bit, but Skerritt's is as big as your hat.'

"Skerritt was genuinely fond of physical and mental hard work. I spent many an hour watching him dip clinkers in a bucket of cement to build them together into a large fernery at Thornton House. He worked harder than any mason could have been induced to do, without coat, waistcoat or collar, and with his sleeves rolled up, and was bubbling over with fun when he was nearly caught by patients in this unclothed condition. He stayed with me in the country at my cottage, and begged to be put to work in the garden. He and I spent many an hour at the back-breaking labour of strawberry planting, and more than once, when I had a mind to ease off for a time, he compelled me by precept and example not to be lazy. He took a deep scientific interest in watching my gardener's attempts to overcome an invasion of 'spot' in tomato plants, and was as delighted as a schoolboy when he, his wife, my wife and I, cut into a peach weighing 17 oz. which my gardener had grown.

"He had a great love of altering the residences which he purchased, and employed no architect to draw his plans. For months he was busy designing and seeing executed the beautiful oak room which served as a waiting-room at Thornton House and at Edgecumbe House, and the beloved fittings of which followed him to the house in which he died, and were there re-erected. In this last place of residence he gratified to the full his architectural talent. Moreover, he undertook personally the improvements in his lovely garden, and discussed with me, as if the matter were one of extreme importance, the selection of apple and pear trees. In the last weeks of his life—in fact, up to the very last day he was able—he was engaged in heavy rock work in the quarry attached to his house, which even involved blasting. This work my dear old friend leaves, like few things to which he put his hand, unfinished.

"Many years ago he and I went to Berlin together to see for ourselves what we could of the value of Koch's Tuberculin. I am bound to confess that I had intended to make this something of a picnic among my old German friends, but I found I was vastly mistaken. Skerritt went to work, and work he did from morning to night, and compelled me to do the same; and in the week we spent there we worked as hard as I have ever done in my life. This was Markham Skerritt, and his powers of endurance were such that it was extremely difficult to keep pace with him. On our return we wrote a joint paper on Koch's treatment of Tuberculosis, which appeared in the *Bristol Medico-Chirurgical Journal* (Vol. VIII.). This we started to compose at 8 p.m., and Skerritt left me in a most dilapidated condition at five the next morning, having written—with a short interlude for supper—right through the night. So stubborn was his iron constitution, that he was able to go home, have his bath, and set about his daily round of work. When in Berlin we stayed in the house of my honoured old friend Sanitätsrat Dr. Patschkowski, and during our stay our hostess celebrated her birthday. I shall never forget seeing Skerritt with a huge bouquet—which we had purchased for the occasion—in his hand, ushered into a room where a dozen German ladies were in the midst of a 'Kaffee Klatsch,' nor at the supper which afterwards took place, hearing him—who knew no German, whilst some of the guests knew little English—endeavour to converse and make a little speech in Latin. I have had extremely kind messages of condolence with his widow from the friends he then made, showing that he has not been forgotten.

"As husband, father and friend, Markham Skerritt was a rare soul. He ever gave more than he wished to receive; he ever gave of his very best. His judgment was sound and unerring, and his desire to help all those who came to him was boundless. Busy as he was, he could always find time to help others. His opinion was expressed with complete independence to friend, doctor and patient alike, and while such independence may be said to come more easily from men in easy circumstances, this, in my opinion, has no bearing whatever on what was a sterling quality of Markham Skerritt's mind. It was a characteristic proper to him by inheritance, and fostered by environment.

"The shock of the death of his only child—coming to a man of his reserved but deep and tender nature—was one from which he never completely recovered. Long after her death his wife and he dined quietly at my house, and more than once in the course of light conversation his eyes filled with tears, and I knew that he was standing by a graveside. Once speaking to him about it, and apologising, as I did, for reopening a wound, his reply was: You have not done that, my friend, because it is always open.'

"On Monday, 29th April, large numbers of us felt that one of the finest types of Christian gentlemen whom it has ever been our happy lot to know left us and joined the great majority."

Geo. W. Russell, speaking of the Ideal Character, remarks: "The man who owns it may be very homely, very insignificant; as the world judges, very uninteresting. But the character itself bears the sign-manual of Heaven, writ large in purity and courage, and gentleness and unselfishness; and the man, by a secret power which he has never realised, leavens the world in which his lot is cast." And further he quotes from Dr. Liddon: "It is by the work of grace in lives such as this that both the church and society are braced and sanctified; it is from such lives that a truer, loftier, more disinterested, sterner, yet withal, most assuredly not less affectionate spirit than that of common men radiates into and elevates an entire generation." [1]

### BIBLIOGRAPHY OF EDWARD MARKHAM SKERRITT.

"On the treatment of Empyema by Lister's Antiseptic Method."—*Brit. M. J.*, 1876, ii. 109.

"On the treatment of Acute Rheumatism."—*Ibid.*, 1877, ii. 104, 133.

"Spontaneous Rupture of the Spleen."—*Ibid.*, 1878, i. 641.

"Complete Obstruction of Intestine by Croupous Inflammation."—*Ibid.*, 1878, ii. 367.

"Acute Tuberculosis."—*Tr. Bristol M.-Chir. Soc.*, 1878, i. 77—80.

"Croton-chloral in Neuralgia."—*Ibid.*, 10—15.

"A case of Abdominal Aneurism, becoming diffused after distal compression."—*Ibid.*, 102—108.

"Subcutaneous Emphysema and Infiltration, with pus following aspiration for pyo-pneumothorax."—*Ibid.*, 112—115.

"Intestinal Perforation from Ulceration, with Abdominal Tumour of obscure origin."—*Ibid.*, 87.

"Facial Paralysis from Cerebral Tumour."—*Ibid.*, 110—112.

"Remarks in discussion on High Temperature."—*Ibid.*, 135—137.

"A case of Complete Obstruction of the Intestine by Fibrinous Exudation, with Latency of Acute Symptoms."—*Tr. Clin. Soc. Lond.*, 1879, xii. 97—102.

"On the Simulation of Ascites in cases of Intestinal Obstruction."—*Ibid.*, 224—228.

"Clinical Evidence against the Contagiousness of Phthisis."—*Bristol M.-Chir. J.*, 1883, i. 48—70.

"On the Conduction of Physical Signs in Diseases of the Lungs."—*Proc. M. Soc. Lond.*, 1885, viii. 55; also *Brit. M. J.*, 1884, ii. 1005.

"Acute Febrile Glycosuria."—*Brit. M. J.*, 1885, ii. 1052.

"Actinomycosis Hominis."—*Internat. J. M. Sc.*, 1887, xciii. 75—88.

"Diseases of the Lungs and Organs of Respiration."—*Year-Book of Treatment*, 1889—96.

[1] *Social Silhouettes*, Smith, Elder & Co., 1906.

" Koch's Treatment of Tuberculosis."—*Bristol M.-Chir. J.*, 1890, viii. (Supplement).
" Oxygen Gas in Acute Respiratory Affections."—*Brit. M. J.*, 1892, i. 269.
" Clinical Lecture on Interlobular Emphysema of the Lungs."—*Ibid.*, 1010.
Presidential Address to the Bristol Medico-Chirurgical Society on " The Teachings of Failure."—*Bristol M.-Chir. J.*, 1892, x. 229—249.
" Caffeine in Diseases of the Respiratory Organs."—*Practitioner*, 1895, liv. 318—322.
Presidential Address to the Bath and Bristol Branch of the British Medical Association on " Some Current Topics in the British Medical Association."—*Brit. M. J.*, 1896, ii. 119—121.
The Bradshaw Lecture on " Prognosis in Heart Disease."—*Ibid.*, 1897, ii. 1327—1332 ; also *Lancet*, 1897, ii. 1163—1167.
" Case of Protracted Sleep extending over fifty days."—*Brit. M. J.*, 1898, ii. 957.
" Hospital Management in Bristol."—*Practitioner*, 1901, lxvi. 671—672.
" Some Points in the Diagnosis and Prognosis of Heart Disease."—*Polyclinic* (Lond.), 1902, vi. 61—67.
" On Cardiac Pain."—*Proc. M. Soc., Lond.*, 1902, xxv. 181—196.

## Figures and Tables

**Figure f1:**